# Factors associated with institutional delivery: Findings from a cross-sectional study in Mara and Kagera regions in Tanzania

**DOI:** 10.1371/journal.pone.0209672

**Published:** 2018-12-26

**Authors:** Dunstan R. Bishanga, Mary Drake, Young-Mi Kim, Amasha H. Mwanamsangu, Ahmad M. Makuwani, Jeremie Zoungrana, Ruth Lemwayi, Marcus J. Rijken, Jelle Stekelenburg

**Affiliations:** 1 Jhpiego Tanzania, Dar es Salaam, Tanzania; 2 University of Groningen, University Medical Centre Groningen, Department of Global Health, Health Sciences, Groningen, the Netherlands; 3 Jhpiego, Baltimore, MD, United States of America; 4 Tanzania Ministry of Health, Community Development, Gender, Elderly and Children, Dar es Salaam, Tanzania; 5 Department of Obstetrics and Gynecology, Division of Woman and Baby, University Medical Center Utrecht, Utrecht, The Netherlands; 6 Julius Global Health, Julius Center for Health Sciences and Primary Care, University Medical Center Utrecht, Utrecht, the Netherlands; 7 Department of Obstetrics and Gynecology, Leeuwarden Medical Centre, Leeuwarden, the Netherlands; Brandeis University, UNITED STATES

## Abstract

In Tanzania, maternal mortality has stagnated over the last 10 years, and some of the areas with the worst indicators are in the Lake and Western Zones. This study investigates the factors associated with institutional deliveries among women aged 15–49 years in two regions of the Lake Zone. Data were extracted from a cross-sectional household survey of 1,214 women aged 15–49 years who had given birth in the 2 years preceding the survey in Mara and Kagera regions. Logistic regression analyses were conducted to explore the influence of various factors on giving birth in a facility. About two-thirds (67.3%) of women gave birth at a health facility. After adjusting for possible confounders, six factors were significantly associated with institutional delivery: region (adjusted odds ratio [aOR], 95% confidence interval [CI]: 0.54 [0.41–0.71]), number of children (aOR, 95% CI: 0.61 [0.42–0.91]), household wealth index (aOR, 95% CI: 1.47 [1.09–2.27]), four or more antenatal care visits (aOR, 95% CI: 1.97 [1.12–3.47]), knowing three or more pregnancy danger signs (aOR, 95% CI: 1.87 [1.27–2.76]), and number of birth preparations (aOR, 95% CI: 6.09 [3.32–11.18]). Another three factors related to antenatal care were also significant in the bivariate analysis, but these were not significantly associated with place of delivery after adjusting for all variables in an extended multivariable regression model. Giving birth in a health facility was associated both with socio-demographic factors and women’s interactions with the health care system during pregnancy. The findings show that national policies and programs promoting institutional delivery in Tanzania should tailor interventions to specific regions and reach out to low-income and high-parity women. Efforts are needed not just to increase the number of antenatal care visits made by pregnant women, but also to improve the quality and content of the interaction between women and service providers.

## Introduction

Maternal mortality is one of the most daunting health challenges facing the world today [1]. Sustainable Development Goal 3 is the reduction of the global maternal mortality ratio to less than 70 deaths per 100,000 live births by 2030 [2]. In Tanzania, which has an estimated population of 56 million, the maternal mortality ratio remains unacceptably high, at 556 deaths per 100,000 live births in 2015–2016 [3]. There has been no notable progress over the past decade despite the existence of proven solutions and increased support at both the national and international levels [3–5].

One important global strategy to reduce maternal and perinatal deaths in low- and middle-income countries, such as Tanzania, is to increase the number of women giving birth in a health facility [6–9]. Currently, less than two-thirds of births in Tanzania take place in health facilities (63%), but the Government of Tanzania aims to reach 80% skilled birth attendance by 2020 [3]. A mid-term review of the national strategic plan to accelerate the reduction in maternal, newborn, and child deaths in Tanzania (2008–2015) showed a disparity in skilled birth attendance coverage across the country’s six zones, with the lowest rates in Western and Lake Zones [6]. The two regions that are the subject of this study, Mara and Kagera, are located in the Lake Zone, in northwestern Tanzania along the western and eastern shores of Lake Victoria. The organization of health services and infrastructure is similar in the two regions, and both have around six health workers per 10,000 population [6]. The regions of the Lake Zone have some of the worst maternal health indicators in the country and have been prioritized by the government for strategic investments and interventions given their poor coverage of key reproductive, maternal, newborn and child health (RMNCH) interventions, such as skilled birth attendance, postnatal care, and family planning [6]. According to the 2015–16 Tanzania Demographic and Health Survey (TDHS), 97% of women in the Lake Zone attended at least one antenatal care (ANC) visit during pregnancy, but the institutional delivery rate was only 49.8% [3].

Researchers have identified several factors that may influence institutional delivery rates and skilled birth attendance, although there is limited information from northwestern Tanzania, particularly in Kagera and Mara. Studies have found an association between institutional delivery rates and socio-demographic factors, including woman’s education, wealth [7–9], residence [8,10,11], and parity [8,10–12]; care during pregnancy, including early antenatal care (ANC) visits [9], number of ANC visits [11,13], ANC messages regarding the importance of institutional delivery [9], and counseling on and/or knowledge of danger signs of pregnancy and labor [12,14]; and practices such as birth preparedness/complication readiness [11,14,15] and decision making on health care [8,9,12,16].

Since 2014, the government has prioritized selected interventions in Kagera and Mara regions to improve maternal health indicators, including ANC attendance, deliveries in health facilities with assistance from skilled providers, access to emergency obstetric and newborn care services, and facility responsiveness to stock-outs of RMNCH commodities [6]. To support targeted interventions, our study investigated factors that are associated with institutional delivery in Kagera and Mara. We examined various socio-demographic variables, utilization and delivery of ANC services, and male involvement. The findings can be useful in guiding the design and implementation of programs and policies to promote facility-based childbirth in northwestern Tanzania.

## Materials and methods

### Study design and setting

In this study, we analyzed a subset of data from a cross-sectional household survey on knowledge, practices, and coverage related to maternal and newborn health, immunization, malaria, and family planning that was conducted in April 2016 by the Maternal and Child Survival Program (MCSP) in Mara and Kagera regions in the Lake Zone of Tanzania. The aim of the survey was to measure the existing maternal, newborn, immunization, and family health indicators for the catchment population in these two regions as a baseline for the project, where MCSP subsequently worked collaboratively with local stakeholders to implement a comprehensive integrated RMNCH program. The study participants were women aged 15–49 years, who had given birth during the 2 years preceding the survey (referred to as recently delivered women in this paper) and were members of the selected households. All study participants from the cross-sectional household survey (1,263) were included in the analysis for this study, except for those women (49) who did not respond to the question on place of delivery.

### Sampling

The knowledge, practices, and coverage household survey used a two-stage, stratified-cluster sampling design. Administratively, regions were divided into districts, which in turn were sub-divided into wards. The Tanzania HIV/AIDS and Malaria Indicator Survey and Population Census wards were further broken down into enumeration areas (EAs), each EA typically include about 100 households. The EAs served as the primary sampling units in the survey. EAs were selected through the probability proportional to size method, within each urban and rural geographical strata, based on the household size information from the latest (2012) population census.

We selected 20 recently delivered women in each EA (fixed take-size of 20) and select 32 EAs in each region. In each EA, the first household was selected at random from the generated household list. Then additional households were systematically selected from a list of households at a calculated set interval using the rural Tanzania general fertility rate in the study area, which was 200 per 1,000 women (the general fertility rate in rural Tanzania is 210 per 1,000 women according to the 2010 TDHS). To determine the interval for selecting households from the list, we divided the total number of households in each enumeration area by 50.

Households were visited in sequence as they appeared in the list until 20 recently delivered women were located, agreed to participate, and were interviewed. If more than one eligible participant in a household consented to participate in the study, all were interviewed. However, the socio-economic status module was administered to only one respondent in the household—ideally the eldest eligible respondent. A total of 1,263 women were interviewed (Kagera = 629; Mara = 634). Of these, 49 women were excluded from this analysis because they did not answer the question on place of delivery, resulting in a final sample of 1,214 women (Kagera = 612; Mara = 602).

#### Data collection

The survey employed a structured questionnaire developed by the Child Survival Health Grants Program and the Maternal and Child Survival Program, which was adapted by RMNCH technical experts to reflect the Tanzanian context. The tool included questions on socio-demographic information, topics covered during counseling (response options included: danger signs during pregnancy, nutrition during pregnancy, rest during pregnancy, self-care during pregnancy, individual birth preparedness, danger signs during delivery, postpartum family planning, postpartum danger signs for mothers, danger signs for the newborn, initiation of breastfeeding, and exclusive breastfeeding), and services received during ANC visits (response options included: blood pressure measured, urine test, blood test, HIV test, TB test, syphilis test, iron tablets/syrup, drugs for intestinal worms, and anti-malarial drugs). After the questionnaire was translated into the Kiswahili language, reviewed by local experts, and pilot-tested with research assistants, some questions were refined to improve their comprehensibility in Kiswahili. Data collection tools were uploaded to the CommCare HQ mobile data collection platform on tablets.

A team of 30 female and male research assistants was recruited and trained on research ethics, the study protocol, household sampling, informed consent, and other data collection procedures. Data were collected in face-to-face interviews conducted in Kiswahili and recorded on password-protected tablets. Inbuilt skip patterns helped assure data quality. In addition, a data manager reviewed the data daily and alerted study supervisors to errors so that they could be addressed immediately.

### Data analysis

The primary outcome of the analysis was the place of delivery for a woman’s most recent birth in the last 2 years. Respondents who reported a home birth (at their own home or another’s) were compared with those who gave birth at a health facility.

Independent variables were selected based on the literature highlighted in the introduction. Socio-demographic variables included each woman’s region of residence, age, education, marital status, number of children, and household wealth. To measure household wealth in a non-cash economy, we used 20 survey questions about the ownership of various items, including: type of toilet, type of house, number of rooms and number of sleeping spaces, source of water supply, existence of an indoor kitchen, source of fuel for cooking and energy, type and number of domestic animals owned by household, nature of transport owned, main economic activity for the household, and ownership of mosquito nets. All socio-economic data were based on self-reports by the mothers or guardians in the house at the time of the survey. We used a principal component analysis as developed by Filmer and Pritchett [17] to calculate a wealth index based on the 20 ownership questions. Ownership questions were considered for the factor if their score contributions to the principal component analysis were between 10% and 90%. Five ranks of wealth index (lowest, lower, middle, higher, and highest) were used to classify the households’ socio-economic status; then the respective factor scores were categorized and used in the regression analysis.

Women’s utilization of the health system was assessed by the self-reported number of ANC visits during their most recent pregnancy; women who made no ANC visits were compared with those making one to three visits and those making at least four visits per national guidelines. Other indicators of the coverage and quality of ANC services were the number of services received during ANC visits and the number of topics on which women were counseled. Finally, we assessed women’s knowledge of danger signs during pregnancy, male partner participation in health care decision making and ANC visits, and the number of preparations made for the birth.

Categorical variables were presented as frequencies and percentages and Pearson’s chi square test was used to test for differences. If the number of participants in one or more categories was less than five, Fisher’s exact test was applied. Results were considered to be significant with a p value of ≤ 0.05. Logistic regression was used to investigate the association between independent variables and outcomes. The final multivariable model included all variables that were statistically significant in a bivariate analysis and that had responses from all participants, in order to maintain the sample size used for the analysis. Thus, variables that only applied to women who had attended ANC (e.g., number of counseling topics covered and services received during ANC) were excluded from the final model. Clustering at the level of the primary sampling units was adjusted by use of Huber-White sandwich errors [18]. In addition to the final multivariable model, we ran an extended multivariable model that includes all variables, regardless of significance in the bivariate analysis and completeness of responses, in order to observe whether there was any difference on determinants of institutional delivery; this model was limited to women who attended ANC. Results were presented as odds ratios with corresponding 95% confidence interval. All analyses were performed using STATA 14 (College Station, Texas, USA, 77845).

### Ethical considerations

All study participants provided oral consent to accommodate various levels of literacy. A research assistants read the consent form aloud in Kiswahili and answered the woman’s questions. If the woman gave her consent to participate, the research assistant wrote down her identification number from a pre-assigned list of identification numbers and signed the form to certify that she gave her consent. For women aged 15–17 years who had a child but was not in union, the consent of a parent or guardian was required in addition to the woman’s own consent for enrollment in the study. The consent procedure and its forms were approved by both IRBs overseeing the study. Interviews were conducted by data collectors in the home in an area that provided privacy and where no one could overhear what was said. This study was reviewed and approved by the Johns Hopkins Bloomberg School of Public Health Institutional Review Board and the Medical Research Coordinating Committee of the National Institute for Medical Research of Tanzania.

## Results

### Socio-demographic factors, ANC attendance, and place of delivery

The 1,214 mothers in the study sample were about evenly divided between Kagera and Mara regions. Half (53.1%) were aged 20–29, more than three-quarters (79.7%) had at least a primary education, most (88.1%) were in union, and most (78.3%) had at least two children (Table 1).

**Table 1:** Bivariate analysis of mother’s socio-demographic characteristics and ANC attendance, by place of delivery (N = 1,214).

Socio-demographic characteristics	Number	Percent distribution (%)	Proportion by place of delivery		P value
			Home	Facility	
**Total**	1,214	100.0	32.7	67.3	
**Region**					
Kagera	612	50.4	24.7	75.3	<0.001
Mara	602	49.6	40.9	59.1	
**Age group**					
15–19	155	12.8	27.1	72.9	0.150
20–24	356	29.3	29.8	70.2	
25–29	289	23.8	33.6	66.4	
30–34	235	19.4	37.0	63.0	
35+	179	14.7	36.3	63.7	
**Education level**					
No formal education	246	20.3	43.9	56.1	<0.001
Primary education	846	69.7	31.6	68.4	
Secondary education or higher	122	10.1	18.0	82.0	
**Marital relationship**					
In union	1,069	88.1	33.3	66.7	0.226
Not in union	145	11.9	28.3	71.7	
**Number of children**					
1 child	263	21.7	20.2	79.9	<0.001
2–4 children	552	45.4	33.2	66.9	
5 or more children	399	32.9	40.4	59.7	
**Household wealth index**					
Lowest quintile	247	20.4	45.3	54.7	<0.001
Lower quintile	239	19.7	36.8	63.2	
Middle quintile	262	21.6	35.1	64.9	
Higher quintile	225	18.5	27.1	72.9	
Highest quintile	241	19.9	18.3	81.7	
**Number of ANC visits attended**					
None	73	6.0	52.1	47.9	<0.001
1–3	506	41.7	39.5	60.5	
4+	635	52.3	25.0	75.0	

ANC, antenatal care.

Two-thirds (67.3%) of the women gave birth in a health facility and the rest at home. A greater proportion of women from Kagera (75.3%) than Mara (59.1%) gave birth in a health facility (p<0.001). The likelihood of delivering in a health facility increased significantly with educational level (p<0.001) and household wealth (p<0.001). As parity increased, the proportion of women giving birth in a facility decreased (p<0.001); 79.9% of women delivering their first child gave birth at a facility, compared with 59.7% of women delivering their fifth or higher child. Neither age nor marital status influenced place of delivery. A small minority of women did not make any ANC visits (6%) and were significantly less likely to deliver in a facility than women who made at least one ANC visit.

### Content and coverage of ANC visits

Most women received counseling on at least five of 11 possible topics (71.6%) and received at least four of nine essential services (72.3%) during ANC visits. Just over half of the women (52.3%) made four or more ANC visits. More than half of the women who attended ANC (61.1%) said their male partner came to at least one ANC visit. Women were significantly more likely to deliver at a facility as the number of counseling topics (p<0.001), services received (p<0.001), and ANC visits (p<0.001) increased, or were accompanied by their male partner to at least one ANC visit (p<0.05) (Table 2).

**Table 2:** Among women who attended ANC, counseling and service coverage and number of visits, by place of delivery (N = 1,141).

Characteristics of ANC services	Number	Percent distribution (%)	Proportion by place of delivery		
			Home	Facility	P value
**Total**	1,141	100.0	31.5	68.5	
Number of topics covered during ANC counseling*					
1–4	324	28.4	42.3	57.7	<0.001
5–8	394	34.5	28.9	71.1	
9–11	423	37.1	25.5	74.5	
Number of services received during ANC visits**					
1–3	179	14.7	50.3	49.7	<0.001
4–6	614	50.6	31.8	68.2	
7–9	348	28.7	21.3	78.7	
**Number of ANC visits made**	** **		** **	** **	** **
1–3	506	44.4	39.5	60.5	<0.001
4 or more	635	55.6	25.0	75.0	
**Husband (male partner) accompanied a woman to at least one ANC visit**					
No	472	38.9	35.2	64.8	0.024
Yes	669	61.1	28.9	71.2	

ANC, antenatal care.

*Counseling topics were: 1) danger signs during pregnancy, 2) nutrition during pregnancy, 3) rest during pregnancy, 4) self-care during pregnancy, 5) individual birth preparedness, 6) danger signs during delivery, 7) postpartum family planning, 8) postpartum danger signs for mothers, 9) danger signs for the newborn, 10) initiation of breastfeeding, and 11) exclusive breastfeeding.

**Potential services were: 1) blood pressure measured, 2) urine test, 3) blood test, 4) HIV test, 5) TB test, 6) syphilis test, 7) iron tablets/syrup, 8) drugs for intestinal worms, and 9) anti-malarial drugs.

### Knowledge of pregnancy danger signs, decision making, and birth preparations

Among women who attended ANC services at least once, only 28.5% of women knew at least three pregnancy danger signs. Substantial numbers of women played no role in making decisions regarding their own health care (44.8%) or where to give birth (33.9%). Birth preparations were common, with 55.3% of women engaging in all four practices (Table 3).

**Table 3:** Knowledge of pregnancy danger signs, health care decision making, husband’s participation, and birth preparations, by place of delivery (N = 1,214).

Knowledge and behaviors	Number	Percent distribution (%)	Proportion by place of delivery		
			Home	Facility	P value
**Total**	1,214	100.0	32.7	67.3	
Number of danger signs during pregnancy that woman knows*					
None	242	19.9	45.5	54.6	<0.001
1–2	626	51.6	32.9	67.1	
3 or more	346	28.5	23.4	76.6	
**Decision maker on health care**					
Woman alone	378	31.1	31.8	68.3	0.021
Jointly (woman and her husband [male partner])	292	24.1	27.1	73.0	
Husband (male partner) alone/others alone	544	44.8	36.4	63.6	
**Decision maker on place of delivery**					
Woman alone	461	38.0	33.8	66.2	0.163
Jointly (woman and her husband [male partner])	342	28.1	28.7	71.4	
Husband alone/others alone	411	33.9	34.8	65.2	
Number of birth preparation components**					
None	211	17.4	74.4	25.6	<0.001
1–3	331	27.3	32.0	68.0	
All 4	672	55.3	19.9	80.1	

ANC, antenatal care.

* The pregnancy danger signs were: 1) vaginal bleeding, 2) fast/difficult breathing, 3) fever, severe abdominal pain, 4) headache, blurred vision, 5) convulsion.

** Components of birth preparations included: 1) saving money, 2) arranging transport, 3) deciding on a birth companion, 4) deciding on a place of delivery.

Facility deliveries were higher for women who had greater knowledge of pregnancy danger signs (p<0.001), participated in health care decision making (p<0.05), or practiced more components of birth preparedness (p<0.001).

### Multivariable logistic regression

After adjusting for all variables, including those related to ANC, five variables remained statistically significant in the extended multivariable logistic regression. Institutional delivery was independently associated with living in Kagera region (p<0.001), giving birth to a first child (p<0.05), being in the highest wealth quintile (p<0.01), making four or more ANC visits (p<0.01), and making birth preparations (p<0.001) (Table 4). However, this model was limited to the 1,141 women who attended ANC.

**Table 4:** Extended multivariable logistic regression of factors associated with institutional delivery among women that attended ANC (N = 1,141).

	aOR [95% CI]	P value
**Region**		
Kagera	Reference	
Mara	0.56 [0.41–0.75]	<0.001
**Highest education level**		
No formal education	Reference	
Primary education	1.34 [0.94–1.93]	0.107
Secondary education or higher	1.59 [0.85–3.01]	0.149
**Marital status**		
In union	Reference	
Not in union	1.02 [0.62–1.66]	0.963
**Number of children**		
1 child	Reference	
2–4 children	0.60 [0.40–0.90]	0.014
5 or more children	0.59 [0.39–0.93]	0.021
**Household wealth index**		
Lowest quintile	Reference	
Lower quintile	1.06 [0.69–1.63]	0.773
Middle quintile	0.96 [0.63–1.46]	0.846
High quintile	1.44 [0.91–2.27]	0.117
Highest quintile	2.08 [1.26–3.43]	0.004
**Number of ANC visits**		
1–3	Reference	
4 or more	1.58 [1.19–2.10]	0.002
**Number of pregnancy danger signs known**		
None	Reference	
1–2	0.94 [0.65–1.39]	0.782
3 or more	1.22 [0.78–1.89]	0.383
**Decision maker on health care**		
Woman alone	Reference	
Jointly	1.11 [0.74–1.67]	0.617
Husband (male partner) alone/ others alone	0.94 [0.65–1.33]	0.711
**Decision maker on place of delivery**		
Woman alone	Reference	
Jointly	0.93 [0.64–1.36]	0.724
Husband (male partner) alone/ others alone	1.02 [0.69–1.43]	0.979
**Number of birth preparation components**		
None	Reference	
1–3 components	6.00 [3.12–11.55]	<0.001
All 4 components	12.17 [6.24–23.73]	<0.001
**Number of topics covered during ANC counseling**		
1–4	Reference	
5–8	1.10 [0.76–1.58]	0.623
9–11	0.96 [0.66–1.42]	0.855
**Number of services received during ANC visits**		
1–3	Reference	
4–6	1.32 [0.88–1.96]	0.180
7–9	1.58 [0.98–2.56]	0.062
**Husband (male partner) accompanied a woman to at least one ANC visit**		
No	Reference	
Yes	0.98 [0.73–1.33]	0.932

aOR, adjusted odds ratio; CI, confidence interval; ANC, antenatal care

The final model included all women, those who attended ANC services as well as those who did not. After controlling for the variables that were significant in the bivariate analysis, six variables remained significant predictors of delivery at a health facility in the multivariable logistic regression. Facility deliveries were more likely to occur among women who: lived in Kagera (p<0.001), were delivering their first child (p<0.05), belonged to the top two wealth quintiles (p<0.01), knew three or more pregnancy danger signs (p<0.001), made at least four ANC visits (p<0.01), and made any birth preparations (p<0.001) (Table 5). Notably, the final model did not include all variables that were significant in the bivariate analysis. Some had to be excluded because only women who attended ANC could provide responses; these were number of topics covered, number of services received, and presence of a male partner during ANC visits.

**Table 5:** Multivariable logistic regression of factors associated with institutional delivery among all respondents (N = 1,214).

Variable	aOR [95% CI]	P value
**Region**		
Kagera	Reference	
Mara	0.54 [0.41–0.71]	<0.001
**Highest education level**		
No formal education	Reference	
Primary education	1.22 [0.86–1.72]	0.259
Secondary education or higher	1.51 [0.82–2.79]	0.191
**Number of children**		
1 child	Reference	
2–4 children	0.61 [0.42–0.91]	0.015
5 or more children	0.57 [0.39–0.92]	0.018
**Household wealth index**		
Lowest quintile	Reference	
Lower quintile	1.02 [0.68–1.53]	0.388
Middle quintile	1.01 [0.67–1.51]	0.334
High quintile	1.47 [1.09–2.27]	0.008
Highest quintile	2.13 [1.33–3.42]	0.002
**Number of ANC visits**		
None	Reference	
1–3	1.21 [0.69–2.12]	0.095
4 or more	1.97 [1.12–3.47]	0.019
**Number of pregnancy danger signs known**		
None	Reference	
1–2	1.34 [0.96–1.86]	0.082
3 or more	1.87 [1.27–2.76]	0.001
**Decision maker on health care**		
Woman alone	Reference	
Jointly	1.10 [0.74–1.64]	0.635
Husband (male partner) alone/ others alone	0.90 [0.65–1.26]	0.555
**Decision maker on place of delivery**		
Woman alone	Reference	
Jointly	0.98 [0.68–1.41]	0.911
Husband (male partner) alone/ others alone	1.05 [0.75–1.48]	0.771
**Number of birth preparation components**		
None	Reference	
1–3 components	6.09 [3.32–11.18]	<0.001
All 4 components	13.21 [7.14–24.46]	<0.001

aOR, adjusted odds ratio; CI, confidence interval; ANC, antenatal care. Adjusted OR: adjusted for region, highest education level, number of children, household wealth index, number of ANC visits, number of pregnancy danger signs known, decision maker on health care and on place of delivery, and number of birth preparations.

## Discussion

Our study shows a significant association between institutional delivery and three socio-demographic variables (region, number of children, and household wealth) and three variables reflecting women’ interactions with the health care system (number of ANC visits, knowledge of pregnancy danger signs, and birth preparations). Our results suggest that the content of ANC visits and male involvement in those visits also have an impact on whether women give birth in a health facility or at home.

Women living in Kagera were more likely to give birth in a health facility than women in Mara. Regional variations in the utilization of maternal health services within countries have been reported in other parts of Africa [8,10,11,19], and experts have urged that these differences be taken into consideration when creating policies and designing programs to improve institutional delivery rates and maternal and neonatal outcomes [19–21]. In Tanzania, Armstrong and colleagues reported subnational variations in care at birth and demonstrated an association between institutional deliveries and workforce and facility density, quality of care, and availability of essential commodities at facilities [22]. While most national strategic documents for maternal and newborn health in Tanzania consider Mara and Kagera to be homogenous parts of the Lake Zone [23], our results suggest that Kagera and Mara may differ on important parameters. Our study shows that even though both are located along Lake Victoria, women from these regions differ when it comes to accessing and using maternal health services. The results suggest a need for more locally targeted interventions to increase facility deliveries. A follow-up study to examine factors contributing to differences between the two regions could provide additional insights.

Women in the top two household wealth quintiles were more likely to give birth in a health facility than those in lower quintiles. Wealth has been associated with institutional deliveries in many low-income countries [10,24,25], and previous studies in Tanzania confirm the link, including the 2015–16 TDHS [3] and a study in one district of Kagera [9]. The impact of wealth is especially important in the Lake Zone, where fewer than 3 people in 10 belong to the highest two wealth quantiles in Tanzania, compared with more than 8 in 10 people in the Eastern Zone [3]. These economic disparities call for national policies and programs promoting facility-based births to take more assertive actions in communities with the greatest needs.

All women, regardless of the number of times they have given birth, are encouraged to deliver in health facilities where they can receive emergency obstetric care, if needed. However, we found that women of higher parity were less likely to give birth in health facilities. This finding is in agreement with other studies in Tanzania and East Africa [3,8]. Although multipara women tend to normalize childbirth and, hence, may be less likely to seek care during labor, we should also consider whether women’s previous interactions with health facilities have an impact. While our study did not measure this, the literature suggests that poor experiences during past deliveries may discourage women from returning for the next birth [9,26,27]. A study in rural, western Tanzania showed that the quality of care during childbirth, including having a respectful provider, influenced women’s decision about whether to deliver at a health facility or at home [28]. Accordingly, improving the experience of care for women during childbirth could encourage them to use health facility services in subsequent pregnancies.

Our study’s findings also suggest that quantity, as well as quality, of women’s interactions with a health facility during pregnancy have an impact on institutional delivery rates. Facility deliveries increased with the number of ANC visits in the multivariable analysis. Accessing ANC, particularly making at least four ANC visits, has been a strong predictor for institutional delivery in various studies [8,10], including in Tanzania [3]. But it is not just the number of visits that matters. To promote “a positive pregnancy experience” with the health system, the World Health Organization recommends that women have at least eight ANC “contacts” that offer meaningful interactions with a provider and serve as a platform for health promotion and education as well as screening and diagnosis [29]. In our study, women were more likely to deliver in a health facility if they knew at least three pregnancy danger signs and if they made birth preparations, both of which are the products of effective health education and promotion during ANC visits. Previous studies in Ethiopia, Uganda, and Nepal have found a similar association [8,11,14,30]. In addition, the bivariate analysis pointed to the importance of other variables that reflect the quality of ANC interactions: the number of services received and the number of counseling topics covered.

A multi-country study of four African countries underscored the important role of ANC service providers in promoting skilled birth attendance [13]. However, limited time with health providers during ANC visits may limit the exchange of information [31,32]. For example, 4 in 10 women in a study in Kenya did not receive counseling on birth preparedness during ANC visits [33], and less than half of women in Tanzania, Ghana, and Burkina Faso were counseled on most danger signs [34]. Our study also revealed substantial gaps in the services and counseling provided during ANC. These findings call for deliberate efforts to ensure that pregnant women receive adequate counseling and services according to standard protocols during ANC visits.

ANC providers can educate women and their families on danger signs and guide them through a process to develop individual birth plans, which can prepare them for an institutional delivery and to make timely decisions to seek health care in case of an emergency. Despite the proven effectiveness of birth preparedness, however, the majority of women in most low-income countries, including Tanzania, do not engage in this practice [8, 14, 35]. In part, this may be due to the poor quality of education and counseling for pregnant women in Tanzania [36, 37] and other low-income countries [31, 34]. Programs should be working on health education and promotion strategies to ensure that all pregnant women are well informed of danger signs so that they can access timely care. They should also encourage women to prepare for an institutional delivery, while recognizing that women may have good reasons to plan for a home delivery instead, such as experiences with poor care or a lack of supplies at the facility [9, 22, 26, 38].

Husbands’ or male partner’s involvement in maternal health services has been demonstrated to have a positive impact on women’s utilization of services and health outcomes, including ANC, labor and delivery, and postnatal care [12, 39–42]. In our study, women were more likely to give birth at a facility if their male partner accompanied them to at least one ANC visit. Similarly, a study in South Sudan, showed that lack of husbands’ support led to poor utilization of ANC services [43]. In Demographic and Health Surveys of 28 developing countries, the most common reason given by women for not delivering in a facility was that a decision maker at the household level—often a man—considered it “not necessary” [7]. Accordingly, maternal health policies and programs should consider men to be an integral part of pregnancy care. Male partner involvement in ANC can inform men about ways to support pregnant woman, including preparations for delivery at a health facility.

Although greater education has been associated with institutional delivery in studies from India, Ethiopia, and other regions of Tanzania [8,10,11], it was not a predictor in this study. One reason may be that the vast majority of women in this study had little or no education; only 10% had secondary education. Mother’s age and marital status also were not associated with the place of delivery in our study, contrary to the findings of other research [10,11]. The homogeneity of the participants in this study (almost 9 in 10 were in union and over 70% were aged 20–34) may have made it more difficult to observe this kind of association.

### Strengths and limitations of the study

Strengths of this study are the use of a stratified-cluster sampling technique and large sample size, which ensured the survey accurately represented the population of women in Kagera and Mara who had delivered in the previous 2 years. Although recall bias could be a limitation, it is likely to be minimal in this study because pregnancy and childbirth are special and memorable events for a mother. We controlled for confounders by applying multivariate logistic regression. Because the design of the study was cross-sectional, the findings demonstrate only associations with no causal inferences and because it was conducted in two regions in Tanzania, the findings should not be generalized to the whole country.

## Conclusion

In an effort to reduce maternal mortality in Tanzania, this study examined factors that influence the place of birth (facility versus home) in two regions of Tanzania with lagging RMNCH indicators. Policymakers and program designers can use the results from these under-studied regions to develop effective interventions for promoting institutional delivery. Our results provide further evidence that giving birth in a health facility is significantly associated with socio-demographic variables. The results suggest that national policies and programs for promoting institutional delivery in Tanzania should consider regional variations, even within supposedly homogenous zones. To reduce maternal mortality, programs must also reach out to lower income and higher parity women to prompt more women in these categories to give birth in health facilities.

Our study also provides additional evidence that programs should also focus on the number and quality of women’ interactions with the health care system and male involvement in ANC. This should lead to improved understanding of the danger signs in pregnancy and the importance of birth preparedness, which can increase institutional deliveries. And by increasing institutional deliveries, Tanzania can drive down its long stagnate maternal mortality rates.

## Supporting information

S1 Appendix Knowledge, practices and coverage (KPC) household survey questionnaire. (ZIP)
