# Supplementary material for: Factors associated with institutional delivery: Findings from a cross-sectional study in Mara and Kagera regions in Tanzania
Source: PLoS One. 2018 Dec 26;13(12):e0209672. doi: 10.1371/journal.pone.0209672 (PMC6306247; doi:10.1371/journal.pone.0209672)
Supplement: S1 Appendix — (ZIP) [file pone.0209672.s001.zip › Study Questionnaires_PONE-D-18-16327_20 Oct 2018/Tanzania KPC Equity Questionnaire_English version.pdf]

PI Name: Mark Kabue
Study Title: Knowledge, Practices and Coverage (KPC) Household Survey for Planning, Monitoring, and Evaluating the Maternal and Child Survival Program (MCSP)- Annex 5: Tanzania
IRB No.: 5931
PI Version No./Date: March 30, 2016

Approved: 15Oct16 **IRB No.**: 00005931

TANZANIA: EQUITY

NO.	QUESTIONS AND FILTERS	CODING CATEGORIES	SKIP
101	What is the main source of drinking water for members of your household?	**PIPED WATER**<br>PIPED INTO DWELLING .... 11<br>PIPED TO YARD/PLOT .... 12<br>PIPED TO NEIGHBOR .... 13<br>PUBLIC TAP/STANDPIPE .... 14<br><br>TUBE WELL OR BOREHOLE .... 21<br>**DUG WELL**<br>PROTECTED WELL .... 31<br>UNPROTECTED WELL .... 32<br>**WATER FROM SPRING**<br>PROTECTED SPRING .... 41<br>UNPROTECTED SPRING .... 42<br><br>RAINWATER .... 51<br>TANKER TRUCK .... 61<br>CART WITH SMALL TANK .... 71<br>SURFACE WATER (RIVER/DAM/<br>LAKE/POND/STREAM/CANAL/<br>IRRIGATION CHANNEL) .... 81<br>BOTTLED WATER .... 91<br><br>OTHER ______________ 96<br>(SPECIFY)	➤ (14–81)<br>➤ (91–96)

102	What kind of toilet facility do members of your household usually use?<br><br>IF NOT POSSIBLE TO DETERMINE, ASK PERMISSION TO OBSERVE THE FACILITY.	**FLUSH OR POUR FLUSH TOILET**<br>FLUSH TO PIPED SEWER SYSTEM .... 11<br>FLUSH TO SEPTIC TANK.. 12<br>FLUSH TO PIT LATRINE .... 13<br>FLUSH TO SOMEWHERE ELSE............................. 14<br>FLUSH, DON'T KNOW WHERE .... 15<br>**PIT LATRINE**<br>VENTILATED IMPROVED PIT LATRINE .... 21<br>PIT LATRINE WITH SLAB (WASHABLE) .... 22<br>PIT LATRINE WITH SLAB (NOT WASHABLE) . . 23<br>PIT LATRINE WITHOUT SLAB/OPEN PIT .... 24<br><br>COMPOSTING TOILET 31	

		BUCKET TOILET .... 41<br>HANGING TOILET/HANGING LATRINE .... 51<br>NO TOILET/BUSH/FIELD .... 61<br><br>OTHER ________ 96<br>(SPECIFY)	 <br>→ 104
103	Do you share this toilet facility with other households?	YES .... 1<br>NO 2	 → 105
104	Including your own household, how many households use this toilet facility?	NO. OF HOUSEHOLDS<br>IF LESS THAN 10 .... [0][ ]<br><br>10 OR MORE HOUSEHOLDS .... 95<br>DON'T KNOW 98	
105	What type of fuel does your household mainly use for cooking?	ELECTRICITY .... 01<br>BOTTLED GAS .... 02<br>PARAFFIN/KEROSENE .... 03<br>CHARCOAL .... 04<br>FIREWOOD .... 05<br>CROP RESIDUALS,STRAW,GRASS .... 06<br>ANIMAL DUNG .... 07<br><br>NO FOOD COOKED IN HOUSEHOLD .... 95<br><br>OTHER ________ 96<br>(SPECIFY)	 <br><br><br><br><br><br><br>→ 107
106	Is the cooking usually done in the house, in a separate building, or outdoors?	IN THE HOUSE .... 1<br>IN A SEPARATE BUILDING .... 2<br>OUTDOORS .... 3<br><br>OTHER ________ 6<br>(SPECIFY)	108
107	Do you have a separate room which is used as a kitchen?	YES .... 1<br>NO 2	

108	What is the main source of energy for lighting in the household?	ELECTRICITY .... 01<br>SOLAR .... 02<br>GAS .... 03<br>PARAFFIN-HURRICANE LAMP .... 04<br>PARAFFIN-PRESSURE LAMP .... 05<br>PARAFFIN-WICK LAMP .... 06<br>FIREWOOD .... 07<br>CANDLES .... 08<br><br>OTHER ________ (SPECIFY) 96	
109	How many rooms in this household are used for sleeping?	ROOMS .... [ ][ ]	
110	How many sleeping spaces such as mats, rugs, mattresses or beds are used in this household?	SLEEPING SPACES [ ][ ]	
111	Does this household own any livestock, herds, other farm animals, or poultry?	YES .... 1<br>NO 2	 113
112	How many of the following animals does this household own?<br><br>IF NONE, RECORD '00'.<br>IF 95 OR MORE, RECORD '95'.<br>IF UNKNOWN, RECORD '98'.<br><br>a) Milk cows or bulls?<br>b) Other cattle?<br>c) Horses, donkeys, or mules?<br>d) Goats?<br>e) Sheep?<br>f) Chickens or other poultry?	a) COWS/BULLS .... [ ][ ]<br>b) OTHER CATTLE .... [ ][ ]<br>c) HORSES/DONKEYS/MULES .... [ ][ ]<br>d) GOATS .... [ ][ ]<br>e) SHEEP .... [ ][ ]<br>f) CHICKENS/POULTRY .... [ ][ ]	

113	Does any member of this household own any agricultural land?	YES .... 1<br>NO 2	 → 115
114	How many hectares of agricultural land do members of this household own?<br><br>IF 95 OR MORE, RECORD '950'.	HECTARES .... [ ][ ].[ ]<br>95 OR MORE HECTARES .... 950<br>DON'T KNOW 998	
115	Does your household have:<br>a) Electricity that is connected?<br>b) A radio in working condition?<br>c) A television in working condition?<br>d) A non-mobile telephone in working condition?<br>e) A computer in working conditions?<br>f) A refrigerator in working condition?<br>g) A battery or Generator for power?<br>h) An iron (charcoal or electricity)	YES / NO<br>a) ELECTRICITY .... 1 / 2<br>b) RADIO .... 1 / 2<br>c) TELEVISION .... 1 / 2<br>d) NON-MOBILE TELEPHONE .... 1 / 2<br>e) COMPUTER .... 1 / 2<br>f) REFRIGERATOR .... 1 / 2<br>g) BATTERY .... 1 / 2<br>h) IRON .... 1 / 2	
116	Does any member of this household own:<br>a) A watch?<br>b) A mobile phone?<br>c) A bicycle?<br>d) A motorcycle or motor scooter?<br>e) An animal-drawn cart?<br>f) A car or truck?<br>g) A boat with a motor?	YES / NO<br>a) WATCH .... 1 / 2<br>b) MOBILE PHONE .... 1 / 2<br>c) BICYCLE .... 1 / 2<br>d) MOTORCYCLE/SCOOTER .... 1 / 2<br>e) ANIMAL-DRAWN CART .... 1 / 2<br>f) CAR/TRUCK .... 1 / 2<br>g) BOAT WITH MOTOR .... 1 / 2	
117	Does any member of this household have a bank account?	YES .... 1<br>NO 2	
118	How far is it to the nearest market place?<br>IF LESS THAN ONE KM, ENTER 00.<br>IF MORE THAN 95 KM, ENTER 95.	KILOMETRES .... [ ][ ]	

119	Does your household have any mosquito nets?	YES.......... 1<br>NO.......... 2	
120	How many mosquito nets does your household have?<br><br>IF 7 OR MORE NETS, RECORD '7'.	NUMBER OF NETS . . . . [ ]	
